# Supplementary material for: Analysis on outsourcing service behavior of rice pest and disease control based on Heckman selection model—A case study of ten counties in Fujian Province
Source: PLoS One. 2021 Jul 20;16(7):e0254819. doi: 10.1371/journal.pone.0254819 (PMC8292009; doi:10.1371/journal.pone.0254819)
Supplement: Attachment — Submitted filename: Response to Reviewers.docx [file pone.0254819.s002.docx]

Dear László VASA, PhD and Reviewers:
 Thank you for your letter and for the reviewers’comments concerning our manuscript entitled “**Analysis on** **Outsourcing Service Behavior of Rice Pest and Disease Control Based on Heckman Selection Model-- A Case Study of Ten Counties in Fujian Province**” (ID: PONE-D-21-12763). Those comments are all valuable and very helpful for revising and improving our paper, as well as the important guiding significance to our researches. We have studied comments carefully and have made correction which we hope meet with approval.

A list of changes and responses to reviews are as follows.

**List of Action**

(1) We have amended paper format according to the PLOS ONE style templates.
(2) The corresponding author used the ORCID id.
(3) We amended the title on the online submission form.
(4) We removed the survey area map and replaced it with a place name table.

**Responds to the reviewer’s comments:**
**Reviewer #1:**

Special thanks to you for your good comments. 
**（1）Response to comment:** “The introduction is not comprehensive; the research questions are not set appropriately, and the context is not explained.”
**Response:** In the introduction, we tried to amend the language expression to make it more logical. And we also added the performance of China's agricultural outsourcing service into the literature review for explaining the context.
**（2）Response to comment:** “Chapters 2, 3, and 4 should be one chapter called "Material and Methods.”, and write more in the analysis chapter”

**Response:** The article merges the chapter 2,3,4 in the original manuscript into the new chapter 3 "Material and Methods". In the new manuscript, the chapter 4 “Model estimation results” presents the empirical results of the model and the results are analyzed.
**（3）Response to comment:** write an analytical, critical, and comprehensive literature review chapter.
**Response:** Thanks to the reviewers’ comment, we have added a literature review chapter, which contains the following parts： “2.1 Theoretical basis of agricultural outsourcing service”； “2.2 Study on the influencing factors of agricultural outsourcing services participation”；“2.3 The influence of agricultural outsourcing services on other production factors”；“2.4 Research gap”(Page3-Page5)

**（4）Response to comment:** - there are no limitations of the research indicated.
**Response:** In the ending of chapter 5 “Conclusion and suggestion” ，we put forward some problems worthy of further study.

**Reviewer #2:**

Special thanks to you for your good comments. 
**（1）Response to comment:** “the category "pest" usually covers all harmful organisms, incl. viruses, fungi, animals, etc. Thus, pests and diseases in the text may be corrected for arthropod pests and diseases.”
**Response:** It is really true as Reviewer suggested that the definition of “pest”is too broad. So we have defined and annotated "pest" according to the reviewer’s suggestion in page 1.

**（2）Response to comment:** “in addition to outsourcing of "pest control" service, a broader outsourcing of "pest management" is more appropriate approach.”
**Response:** Thank you for your suggestions. According to some scholars,“pest control “maybe used more frequently (such as “Chen Pin, Zhong Funing, Sun Dingqiang. Farming Time Delay, Yield Loss, and Outsourcing Service Utilization under Labor Shortages: Taking Rice Pest Control as An Example [C], Chinese Conference on Agricultural Forestry Economics and Management (2017CAFEM)”and “Ying R Y,Xu B.Effects of Specialized Crop Pest and Disease Control Services on The Intensity of Pesticide Application[J].Population Resources and Environment in China,2017(08):90-97.”). So we more likely to use “pest control”.
**（3）Response to comment:** “The Authors may add some sentence to the discussion/conclusion on how the outsourcing service may contribute to broader IPM implementation, sustainable production, farming development.”
**Response:** In the chapter5“Conclusion and suggestion”(Page.18）,we added further explanations about how the outsourcing service may contribute to broader IPM implementation, sustainable production, farming development.

We tried our best to improve the manuscript and made some changes in the manuscript.  These changes will not influence the content and framework of the paper. And here we did list the changes and marked in red/blue in revised paper.

We appreciate for László VASA, PhD and reviewers warm work earnestly and hope that the correction will meet with approval.
 Once again, thank you very much for your comments and suggestions.

LinPing Wang

06.14.2021
